# Supplementary material for: Development of chemokine network inhibitors using combinatorial saturation mutagenesis
Source: Commun Biol. 2025 Apr 3;8:549. doi: 10.1038/s42003-025-07778-6 (PMC11969024; doi:10.1038/s42003-025-07778-6)
Supplement: Supplementary file 4 — Supplementary Data 2 [file 42003_2025_7778_MOESM4_ESM.zip › SupplementaryData3/scripts/xattune_dr.tar!/./script/analysis/JK/JK_E221_INSULITIS_ATC_plot.pdf]

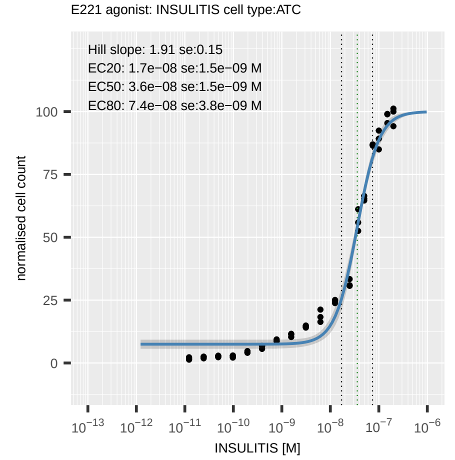

E221 agonist: INSULITIS cell type:ATC
Hill slope: 1.91 se:0.15
EC20: 1.7e−08 se:1.5e−09 M
EC50: 3.6e−08 se:1.5e−09 M
EC80: 7.4e−08 se:3.8e−09 M
normalised cell count
100
75
50
25
0
10^{-13} 10^{-12} 10^{-11} 10^{-10} 10^{-9} 10^{-8} 10^{-7} 10^{-6}
INSULITIS [M]
